# Considering the lip print patterns of Ibo and Hausa Ethnic groups of Nigeria: checking the wave of ethnically driven terrorism

**DOI:** 10.1186/s40163-023-00183-6

**Published:** 2023-03-02

**Authors:** G. C. Uzomba, C. A. Obijindu, U. K. Ezemagu

**Affiliations:** Department of Anatomy, Faculty of Basic Medical Sciences, College of Medical Sciences, Alex Ekwueme Federal University Ndufu Alike, Abakaliki, Ebonyi State Nigeria

**Keywords:** Heterogeneous population, Forensic investigation, Dermal glyphic, Biafra

## Abstract

**Introduction:**

Lip print of an individual is distinct and could be a useful form of evidence to identify the ethnicity of a terrorist.

**Objectives:**

The study analyzed the distribution of lip print patterns of two major ethnic groups in Nigeria; Ibo and Hausa, to develop a strategic plan to check the wave of ethnically driven terrorism in Nigeria, carried out by groups such as Boko Haram and Indigeneous People of Biafra (IPOB).

**Materials and methods:**

The study comprised 800 participants of Ibo and Hausa ethnic groups (400 males and 400 females). The study adopted a digital method of lip print analysis and followed the guidelines outlined by the Institute of Medicine (IOM) for anthropometric measurements. The lip was classified, using Tsuchihashi and Suzuki method of classification.

**Results:**

The predominant lip print patterns of Ibo were Type I with complete vertical groove and Type III with intersect of groove for male and Type III for female. Type I’ with partial length groove was the predominant pattern for both male and female Hausa. The lip width and height of female Ibo were longer than that of the Hausa counterpart (P < 0.05), but none of the anthropometric variables could predict the lip print pattern.

**Conclusion:**

The lip size and print could aid forensic investigation, though genetic diversity and heterogeneity of ethnic groups in Nigeria, especially that of Ibo, could setback use of lip print pattern to identify the ethnicity of an unknown individual in Nigeria to help determine the terrorist group to which they may belong.

## Introduction

In Northern Nigeria, Islamic State—*Boko Haram* that spearheads terrorism in Nigeria are predominantly Hausa, while in Southern Nigeria, Indigenous People of Biafra (IPOB) are predominantly Ibo that want to violently secede from Nigeria. The spate of terrorism in Nigeria is overwhelming the security formation and tactics, and sometimes *Boko Haram* and IPOB gain control of some territories in north-east and south-east Nigeria, respectively. The new public order to wear a facemask due to Corona virus pandemic invalidates some of the forensic methods to identify a criminal, using facial appearance and expression. Most biometric techniques to identify a culprit, such as DNA and fingerprint analysis are not available or are too costly. Therefore, there is a need to explore other new methods to curb the burden becomes expedient. The wave of terrorism in northern and southern Nigeria follows an unpredictable pattern and is becoming more deadly (Nnam et al., 2020). It is multifaceted, but identifying the ethnicity/tribe of the culprits could enhance a strategic plan to check how it ravages the society properly (Strategic Framework for Countering Terrorism and Targeted Violence, 2019).

It is noteworthy that terrorists in Nigeria use religion and ethnicity to divide communities, incite violence and commit atrocities, such as suicide bombings, kidnapping, armed robbery and shooting of innocent citizens (Akinsola & Ojo, 2015). *Boko Haram* are known to bomb churches and schools, and to kidnap wealthy individuals for ransom and women to carry out suicide bombing. In recent years, biometrics techniques for identifying offenders have become more reliable and widely used in forensic investigation (Adamu, 2016; Goonerathne, 2011; Kasprazak, 2000), especially, in personal recognition and criminalization (Vahanwala & Parekh, 2000). Nowadays, closed-circuit televisions are mounted in markets, events and religious centers for video surveillance of criminals. Video surveillance and photographs of individuals capturing the lips can reveal to law enforcement agencies the lip prints, cosmetics used and pathological changes of the lips of individuals at the scene of a crime. Lip print is an evolving forensic technique that is more cost effective and quicker than DNA and fingerprint methods (Kapoor & Badiye, 2017; Simovic et al., 2016) that require High Tech which are not affordable in Nigeria. Often, criminals disguise themselves with face masks and hand gloves, exposing only their eyes and lips while perpetrating atrocities. Lip print pattern can be identified at a glance and if linked to tribes, it could enhance counterterrorism strategies and proactive measure to check ethnically driven terrorism. We considered the lip print pattern as a forensic tool to identify the ethnicity of individuals in Nigeria, with the aid of a camera.

Lip print is genetically determined and is often assumed to be characteristic to an individual (Standring, 2005), but note the advice from the US National Research Council (2009) that is not possible to say conclusively that that any personal feature is unique. It is an indelible mark produced by the natural lines and wrinkles in the vermillion border zone (Augustine et al., 2008; Coward, 2007; Dineshshankar et al., 2013). It is recovered even after alterations such as inflammation, trauma and herpetic lesions (Tsuchihashi, 1974; Tsuchihashi & Suzuki, 1970). Studies have been conducted in different parts of the globe to determine the predominance of lip print patterns for sex identification (Koneru et al., 2013; Kundu et al., 2016; Randhawa et al., 2011). Forensic investigators may use lip prints along with fingerprints, facial expression and dental eruption to detect ethnic differences, age and sex among offenders (Adamu et al., 2015; Ezemagu et al., 2018; Babel et al., 2013). Trace of physical evidence at the crime scene such as a shirt, handkerchief, cutlery and biological materials can assist in forensic crime detection, but identifying an unknown person or criminal properly, has been challenging (Kavitha et al., 2009; Sharma et al., 2010; Vats et al., 2012). The ethnic diversity of Nigerian population with distinct geographical and ecological conditions of northern and southern Nigeria requires a definite protocol to identify and document the ethnicity of her citizens. Unfortunately, documented evidence comparing the lip print patterns of these tribes is not readily available in public domain.

There is need for such documents to help form part of strategies to curb the spate of ethnically driven terrorism in Nigeria, especially now that there is high level of insecurity and internal migration in search of safety and healthy living conditions. Therefore, the current study aims to categorize the lip print patterns of Ibo and Hausa ethnic groups in Nigeria, and also determine the sexual differences in lip print patterns. The study could be useful in identifying the ethnicity of an unknown terrorists in Nigeria.

## Materials and methods

### Participants

A sample size of eight hundred (800) participants (age range 18–55 years) was considered in the study. The age range takes in around 65% of the Nigerian population, and covers the ages when most criminal activity is carried out. The subjects in the sample size were distributed across two major ethnic groups in Nigeria base on tribe and gender; Hausa (200 males; 200 female) and Ibo (200 male; 200 female). The study was carried between January to June, 2021. This cross sectional research study adopted a convenience sampling technique which gives 5% margin of error and 95% level of confidence for quantitative analysis, and accounts for subjects that are more readily accessible in the population. The Ethics and Research committee of Alex Ekwueme Federal University Ndufu Alike considered and approved the study, in line with the requirements of Helsinki’s declaration on human subjects (REF NO: AEFUNAI/VOL/RE/FBMS/024). Only those who gave informed verbal consent and are of Hausa and Ibo origin and whose lips show no pathological signs or congenital anomalies were allowed to participate in the study. The data collection was carried out by the authors and two research assistants who could communicate in Hausa and Ibo languages, fluently. The experiment was carried out twice to ensure that we had accurate data.

### Method

**Fig. 1:**
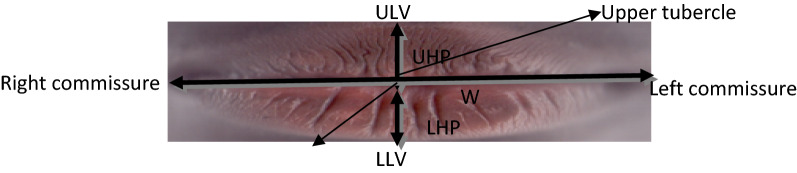
Illustration of the measurement of Lip height and width

**Fig. 2:**
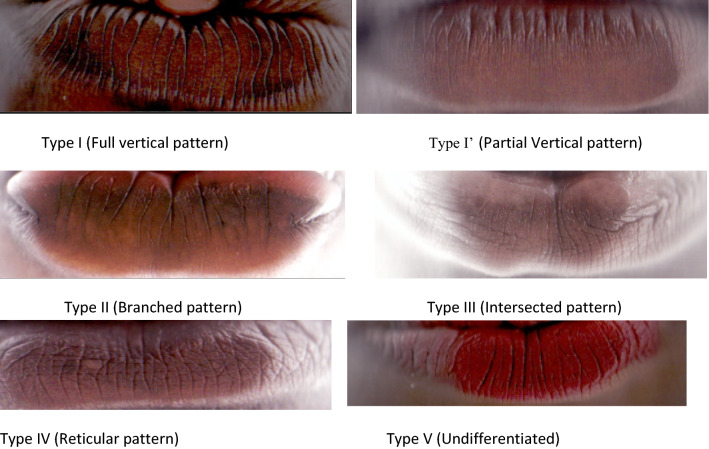
Classification of lip print patterns of Nigerian Ibo and Hausa ethnic groups, using Tsuchihashi and Suzuki method of lip classification (1970)

The height and weight of each subject were measured using a health scale (model RGZ-160, England) and recorded to the nearest 0.1 kg and 0.1 m respectively. The subjects were made to stand at erect position without support, looking at Frankfurt plane whilst their weight and height were measured (Ezemagu et al., 2021; Goons et al., 2011). Regarding lip print, the subject was made to stand in a relaxed position and the lips were cleaned with a soft and comfortable wipes for easy visibility. The photo scanner (HP, HQ-TRE 71,025 with a resolution of 1200 dpi, Boeblingen, Germany) and HP laptop were properly connected and placed on a table where there was illumination for clear appearance of the patterns of lip print. Thereafter, the subject was made to gently bend towards the photo scanner for the lip to be scanned (Ezemagu et al., 2018). The scanned images were cropped, inverted in red scale and was further divided into equal grids to measure the height and width of the lips, using the software calibrated rule. The height and width of the lips were measured as shown in Fig. 1. Thus, the height of upper lip and lower lip was measured as a vertical line between upper lip vermillion (ULV) and lower lip vermillion (LLV) borders passing through central tubercle of upper lip. The width was measured as a transverse line from left to right oral commissures. The scanned image was transferred and stored in the laptop and the scanner cleaned with a sanitizing wipe and allowed 10 s to dry for another use. The lip print pattern was traced and classified using Tsuchihashi and Suzuki method of classification (Tsuchihashi & Suzuki, 1970); Type I, Type I’, Type II, Type III, Type IV and Type V. Lip print with clear-cut groove running vertically across the lip was classified as Type I pattern, and those with partial-length groove of Type I as Type I’. Lip print with branched groove, which partially, resembles the shape of Y alphabet was classified as Type II pattern, and those with intersect of grooves in form of X alphabets as Type III pattern. Lip print with intersect of grooves showing a reticular pattern was classified as Type IV, while those showing multiple interconnected grooves and could not resemble Type I to IV were considered as Type V pattern, as shown in Fig. 2.

### Statistical analyses

Descriptive statistics of the lip print patterns and lip height and width parameters of the participants were carried out and the significance of mean difference between male and female features was tested by two sample *t*-test. The prevalence of lip print patterns in the sample of Hausa and Ibo males and females was expressed in percentage, and chi-square was used to test the significance of the frequency distribution and gender difference. Logistic regression was performed to ascertain the anthropometric features that could predict the lip print patterns. The result is significant when p < 0.05 (95% confidence interval and 0.05 error margin). Data were analyzed with the aid of SPSS version 23.0 (IBM, Armonk, NY, USA).

## Results

**Fig. 3:**
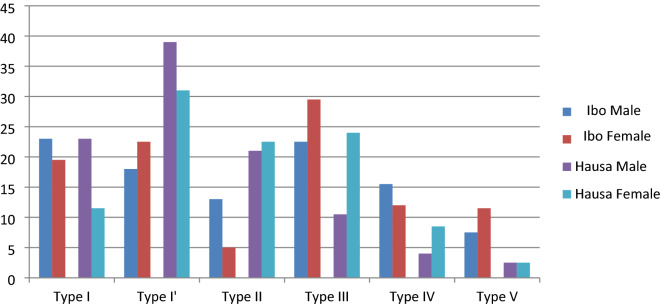
shows frequency distribution of lip print patterns of male and female Hausa and Ibo ethnic groups of Nigeria

Table 1 shows that body height, lip width and height of female Ibo are higher than that of female Hausa in the population *(P* < *0.05)*. Table 2 shows that body height, lip width and height of male Ibo are greater than that of female Ibo. It also shows that all the variables of Hausa male were more than that of female Hausa *(P* < *0.05).* Table 3 shows that Type I (23.5%) and III (22.5%) were predominant lip print patterns for male Ibo and Type III (29.50%) for female Ibo, while the predominant lip print pattern for both male and female Hausa was Type I’; 39.00% and 31.00%, respectively. However, the frequency distribution was not statistically significant *(P* > *0.05).* Although, the data were distributed normally, with equality of variance in lip print pattern among male and female Hausa and Ibo ethnic groups of Nigeria, the logistic regression model in Table 3 could not predict lip print pattern for sex and tribe. Figure 3 shows frequency distribution of lip print patterns of male and female Hausa and Ibo ethnic groups of Nigeria.

**Table 1:** Descriptive statistics of anthropometric parameters of male and female Ibo and Hausa ethnic groups of Nigeria

Parameters	Male		Female		Ibo M v Hausa M	Ibo F v Hausa F	Ibo M v Ibo F	Hausa M v Hausa F
	Ibo	Hausa	Ibo	Hausa				
Age (years)	23.50 ± 6.57	26.69 ± 8.42	24.07 ± 6.75	23.99 ± 8.78	< 0.001	0.936	0.393	0.002
Weight (kg)	56.69 ± 12.71	64.97 ± 12.56	56.66 ± 11.07	57.01 ± 15.10	< 0.001	0.568	0.980	< 0.001
Height (cm)	164.19 ± 10.02	168.58 ± 7.67	157.91 ± 6.59	156.13 ± 8.74	< 0.001	0.024	< 0.001	< 0.001
Lip height (cm)	2.41 ± 0.63	2.45 ± 0.70	2.18 ± 0.52	1.93 ± 0.87	0.491	< .001	< 0.001	< 0.001
Lip width (cm)	6.51 ± 0.66	6.96 ± 0.61	6.34 ± 0.52	5.90 ± 0.63	0.306	< .001	0.028	< 0.001

Table 1 shows a significance mean difference in age, body weight and height of male Hausa and Ibo and body height, lip width and height of female Hausa and Ibo *(P* < *0.05)*, but not for lip height and width of male and body weight and age of female Hausa and Ibo. It also shows a significance mean difference in body height and lip height and width of male and female Ibo, but not for age and body weight. All the variables of Hausa male and female show significance mean difference *(P* < *0.05)*

**Table 2:** Chi square tests for percentage distribution of lip print patterns of male and female Ibo and Hausa ethnics groups of Nigeria

Pattern	Ibo					Hausa				
	Male	Female	Total	Χ^2^	p-value	Male	Female	Total	Χ^2^	p-value
Type I	23.5	19.5	21.50	24.23	0.235	23.00	11.50	17.25	27.24	0.344
Type I’	18.0	22.5	20.25			39.00	31.00	35.00		
Type II	13.0	05.0	09.00			21.00	22.50	21.75		
Type III	22.5	29.5	26.00			10.50	24.00	17.25		
Type IV	15.5	12.0	13.75			04.00	08.50	06.25		
Type V	07.5	11.5	09.50			02.50	02.50	02.50		

Table 2 shows that Type I (23.5%) and III (22.5%) were predominant lip print patterns for male Ibo and Type III (29.50%) for female Ibo, while the predominant lip print pattern for both male and female Hausa was Type I’; 39.00% and 31.00%, respectively. However, the frequency distribution was not statistically significant *(P* > *0.05)*

**Table 3:** Logistics Regression Analysis of the lip print patterns of male and female Ibo and Hausa Ethnic groups of Nigeria

Gender	Parameters	Igbo				Hausa			
		Regression Coefficient (B)	P- value	OR	CI of 95%	Regression Coefficient (B)	P- value	OR	CI of 95%
Male	Age (years)	0.007	0.968	1.007	0.730–1.388	0.199	0.388	1.221	0.777–1.918
	Weight (kg)	0.168	0.556	1.182	0.677–2.065	0.316	0.083	1.372	0.960–1.960
	Height (m)	− 0.169	0.387	0.845	0.576–1.238	− 0.211	0.131	0.810	0.616–1.065
	Lip height (cm)	− 1.834	0.276	4.869	0.282–8.417	0.012	0.071	1.234	0.534–1.123
	Lip width (mm)	1.583	0.395	0.160	0.002–1.094	0.436	0.081	1.677	0.734–1.243
Female	Age (years)	0.188	0.302	1.207	0.845–1.725	− 0.110	0.843	0.896	0.304–2.645
	Weight (kg)	− 0.148	0.335	0.862	0.638–1.166	0.118	0.484	1.125	0.809–1.564
	Height (m)	− 0.042	0.658	0.959	0.797–1.154	0.005	0.959	1.005	0.842–1.199
	Lip height (cm)	2.130	0.317	8.413	0.129–5.467	0.275	0.342	0.765	0.765–1.234
	Lip width (mm)	− 2.368	0.490	0.094	0.001–7.804	− 1.032	0.453	0.661	0.621–2.331

Neither the logistic regression model nor any of the variables could independently predict lip print pattern of male and female Hausa and Ibo, significantly* (P* > *0.05)*

## Discussion

Variation of lip prints in an ethnic group could be a characteristic feature of that population, though genetic diversity between different populations has been a challenge in forensic investigation. The study adopted simple percentage and regression base analyses to quantify the relevance of lip print pattern in identifying the natives of two ethnic groups in Nigeria. The study did attempt to validate the use of lip print patterns to differentiate the ethnicity of individuals from the two major populations of Nigerians, where terrorism is endemic. It also provided baseline data on lip print patterns among the ethnic groups. However, when none of the variables could predict the lip print pattern of Ibo and Hausa, the statistical analysis to develop a protocol for identifying an individual from the two ethnic groups was setback. Nonetheless, the result reveals that lip width and height of female Ibo are significantly higher than that of female Hausa and that lip height and width of male Ibo and Hausa are higher than that of the female counterpart (Table 1).

Although it would be considered rare for individuals to have the same lip print (Durbakula et al., 2015; El Domiaty et al., 2010; Multani et al., 2014), and there was no significant difference in the frequency distribution of lip print patterns, clear patterns were prevalent in male and female Hausa and Ibo populations (Tables 3). We observed that the most predominant pattern in both male and female Hausa was type Type I’, while that of male Ibo was type I and III, and female Ibo was type III. Researchers (Manal et al., 2016) showed type III as predominant type in both Egyptian and Malaysian populations. The findings of Ahmed et al. (2018) and Ghimire et al. (2014) on Egyptian and Dharan populations aligned with the findings of the present study in male Ibo lip print pattern. In a similar vein, Multani et al. (2014); Costa and Caldas (2012) and Simovic et al. (2016) reported type III as the most predominant pattern in male lip prints. Type V (undermined) was the least observed in both tribes and sex. Similar findings were reported by several studies (Manal et al., 2016; Neo et al., 2012; Rao & Kiran, 2016). However, Bindal et al. (2014) reported contrarily on Indian population with the same Tsuchihashi classification. Their study showed that type II was the most predominant pattern in both sex while type III was the least observed in all quadrants. There was a significant difference between lip height and width in both sex and ethnic groups. It was in line with the studies of Ezemagu et al. (2018) and Okeke et al. (2020) who reported a gender difference in the lip print pattern among Ikwo students and Ibo in Anambra State respectively. The findings reveal sexual dimorphism in lip and suggested that ethnicity of an individual can be identified, using lip print.

Furthermore, the study of Ezemagu et al. (2018) revealed that Type I’ is the most predominant lip print pattern among male Ibo students of Alex Ekwueme Federal University, and Okeke et al. (2020) also reported Type IV (28.5%) and Type II (40.0%) as predominant types in male and female Ibo in Anambra state. The results indicate that lip print patterns of Ibo population are inconsistent, which underscores its relevance in personal identification and forensic investigation of criminals in Nigeria. Conversely, the dominant lip print pattern of Hausa population was similar with the findings of Suriya et al. (2019) who reported Type I’ (30.28%) as the most dominant lip print pattern of Deutero-Mala population in Indonesia. This is also supported by the findings of Jeergal et al. (2016) who recorded Type I’ pattern as the most dominant type of lip print in Mangalore population. It was attributed to the presence of Klein zone which forms Type I and Type I’ lip print patterns (Caldas et al., 2007).

Notably, the lip print pattern depends on genetic and phenotypic makeup of the population (Caldas et al., 2007). The logistic regression reveals that anthropometric parameters could not predict the lip print patterns. Similarly, Ezemagu et al. (2018); Okeke et al. (2020) and Nagrale et al. (2014) suggested that lip print of an individual does not depend on his/her anthropometric features, environment, climatic condition and agricultural activities, and that it remains permanent once formed. However, the heterogeneity of major sub-population of Nigeria; Ibo, Hausa and Yoruba, and inter-tribal marriage and bridge cultural practices could have affected the genetic and phenotypic constitutions of the general population. Therefore, comparing the consistent prevalence of each lip print pattern in a population could serve as a measure of its genetic diversity, and factors that promote genetic diversity in a heterogeneous population could setback use of lip print pattern in forensic investigation of criminals.

## Conclusion

The present study reveals the commonest lip print pattern of Ibo: male Type I and III, and female Type III, and Hausa: Type I’ for both sex. It also reveals that Ibo and Hausa has no mutually exclusive distribution of lip print pattern which means that each ethnic group has a combination of the lip print patterns. Furthermore, it reveals that anthropometric features could not predict the lip print pattern of Ibo and Hausa ethnic groups, which suggests that genetic diversity and heterogeneity of the two ethnic groups, especially, that of Ibo population could setback use of lip print pattern to identify the ethnicity of an unknown *Boko Haram* or IPOB terrorists in Nigeria.

## Recommendation

Proper identification of lip print patterns of Nigerian populace with state of the art facility could foster forensic crime investigation, and enhance a strategic plan to check how terrorism ravage the society.

## Data Availability

The authors confirm that all data are fully available without restriction. The entire relevant data are within the manuscript.
